# ^1^H Nuclear Magnetic Resonance Study of Olive Oils Commercially Available as Italian Products in the United States of America

**DOI:** 10.3390/nu4050343

**Published:** 2012-05-04

**Authors:** Laura Del Coco, Francesco Paolo Schena, Francesco Paolo Fanizzi

**Affiliations:** 1 Cancer Research Center, C.A.R.S.O. Consortium, Prov.le Casamassima Valenzano, Km 3, Bari 70010, Italy; Email: laura.delcoco@unisalento.it (L.D.C.); fp.schena@nephro.uniba.it (F.P.S.); 2 Department of Biological and Environmental Science and Technology, Di.S.Te.B.A., University of Salento, Prov.le Lecce-Monteroni, Lecce 73100, Italy

**Keywords:** NMR spectroscopy, extra virgin olive oil, U.S. market oils, food origin characterization

## Abstract

Multivariate analysis of ^1^H NMR data has been used for the characterization of 12 blended olive oils commercially available in the U.S. as Italian products. Chemometric methods such as unsupervised Principal Component Analysis (PCA) allowed good discrimination and gave some affinity indications for the U.S. market olive oils compared to other single cultivars of extra virgin olive oil such as Coratina and Ogliarola from Apulia, one of Italy’s leading olive oil producers, Picual (Spain), Kalamata (Greece) and Sfax (Tunisia). The olive oils commercially available as Italian products in the U.S. market clustered into 3 groups. Among them only the first (7 samples) and the second group (2 samples) showed PCA ranges similar to European references. Two oils of the third group (3 samples) were more similar to Tunisian references. In conclusion, our study revealed that most EVOO (extra virgin olive oils) tested were closer to Greek (in particular) and Spanish olive oils than Apulia EVOO. The PCA loadings disclose the components responsible for the discrimination as unsaturated (oleic, linoleic, linolenic) and saturated fatty acids. All are of great importance because of their nutritional value and differential effects on the oxidative stability of oils. It is evident that this approach has the potential to reveal the origin of EVOO, although the results support the need for a larger database, including EVOO from other Italian regions.

## 1. Introduction

One of the key features of the diet is the nature of fats consumed. Since the 1950s, medical and nutritional investigations confirmed the importance of olive oil, with a high content of monounsaturated fatty acids, for cardioprotective effects and protection against hypertension, diabetes and risk of cancer at several body sites [1,2]. The consumption of virgin olive oil, which is defined as oil obtained only by mechanical extraction from the fruit *Olea europea* L., is increasing due to its nutritional properties stemming from the high content of mono and polyunsaturated fatty acids (oleic, linoleic and linolenic acids) [3]. The fine composition of olive oil, and therefore its sensory characteristics, in addition to being strongly dependent on the nature of the cultivar used for its production, is also influenced by several other factors like climatic and edaphic conditions as well as agricultural practices. Recognition of the influence of these factors has led researchers to study the olive oil obtained from the same cultivar in different geographical areas, over the course of several subsequent harvesting campaigns and at different degrees of olive ripeness [4,5]. In the Mediterranean area, where olive growing has a strong historical, cultural, and economic value, olive oil production and the different analytical methodologies to evaluate the factors that might influence its chemical composition have long been studied [6]. Much data on the characterization of olive oil quality, including single cultivar, produced in Italy, has been described in the literature [7,8,9,10]. The International Olive Oil Council (IOOC) established the definitions and classes of different olive oils (such as methods of production and free acidity of the oil) as well as the rules for their commercialization. Only virgin and extra virgin olive oils (VOO and EVOO) are commercialized on the international markets [11]. According to this Council, world olive oil consumption has risen from 2.8 million tons (1991–1992) to 3.5 million tons (2005–2006), due to the increase in consumption of health foods in many countries, including the United States [12,13]. 

In this paper we report results obtained by unsupervised Principal Component Analysis (PCA) on ^1^H NMR data of blend olive oils commercially available in the U.S., such as Italian products. PCA has been applied to spectral data to reduce the dimensionality of the complex dataset, and to obtain an easy visualization of any clustering or similarity of the various samples. This approach has been successfully used to provide some affinity indications (any sample clustering observed in the PC score plot) for the U.S. market with respect to other EVOO obtained from a single cultivar such as Coratina and Ogliarola from the leading producer Apulia region (Italy) [14], Picual from Spain, Kalamata from Greece and Sfax from Tunisia.

## 2. Experimental Section

Olive oil collection. The Montalbano Agricola Alimentare Toscana S.p.A. provided a collection of commercial olive oils (12 samples) [15]. Olive oils were stored in sealed dark glass bottles labeled with the laboratory code and kept at room temperature in the dark prior to the NMR analysis at the C.A.R.S.O. Consortium. A detailed description of each sample, based on the product label is reported in Table 1. Twenty-five single cultivar olive oil samples, obtained from Apulia (Italy) (5 Coratina samples from the district of Bari-Andria, and 5 Ogliarola samples from the district of Bari-Bitonto), Greece (5 Kalamata samples), Spain (5 Picual samples), Tunisia (5 Sfax samples) were provided by the Basile Azienda Olearia [16]. All samples were collected during the campaign 2009–2010 (Table 1). 

**Table 1:** List of samples. Letter Mand the sequence number are the laboratory code used for samples. Characteristics of the samples and type of merchandising are reported.

Samples Code	Characteristics	Merchandising
M1	Italy 100%	packed
M2	Italy 100%	packed
M3	Italy 100% (Sicily-Apulia)	packed
M4	EVOO-Extracted from Mediterranean Olives-Packed in Italy	packed
M5	EVOO Product of Italy	packed
M6	Italy 100% (Sicily)	packed
M7	EVOO Product of Italy	packed
M8	EVOO Product of Italy	packed
M9	Italy 100%	packed
M10	Italy 100%	unpacked
M11	Italy 100%	packed
M12	Italy 100%	unpacked

### 2.1. NMR Spectra Acquisition

NMR olive oil samples were prepared by mixing 50 µL of the oil sample with 700 µL of deuterated solvent (CDCl_3_). NMR spectra were obtained at 25 °C by the Bruker Avance DRX 500 spectrometer equipped with an inverse triple resonance, z-gradient probe. For each olive oil sample a 1D ^1^H-NMR spectrum was acquired. Spectra were obtained using the following conditions: zg pulse program, 64 K data points (TD) over a spectral width of 14.0019 ppm, 90 degree pulse of 8.40 µs (p1), −0.80 db of power level (pl1), 64 repetitions (NS), relaxation delay of 1 s, T 25 °C.

### 2.2. Characterization of Esterified Fatty Acids by ^1^H NMR Analysis

The percentage of fatty acids, at fatty acids class level, was determined using the integral of proper selected resonances. Resonances were assigned on the basis of the literature data [17]. Intensities were normalized assuming the area of the sn-1,3 signal of triacylglycerol as internal standard [18]. Signal of triplet due to methyl group at 0.95 ppm was used for linolenic acid quantification. As recently reported by Mannina et al., the signal for one of the ^13^C satellites of methyl group belonging to oleic fatty chain appeared in the same region of the linolenic acid triplet, when NMR experiments were performed at high magnetic field (500 MHz) [19]. The correct integral measurement of the linolenic acid triplet was calculated by subtracting the integral of the upfield symmetric oleic ^13^C satellite. 

### 2.3. Statistical Analysis

Multivariate statistical analysis and graphics were obtained using the Amix 3.8.7 (Bruker, Biospin) and the Open Source statistical package R, version 2.11.1 [20]. For multivariate statistical analysis of the bucket-reduced NMR spectra, a PCA procedure was used. Principal component analysis is a way of identifying patterns which highlight the similarities and/or differences between samples and gives an overview of the multivariate profiles. The PCA works by decomposing the X-matrix (buckets linked with the NMR signals) as the product of two smaller matrices (loading and score matrices). 

### 2.4. Data Analysis

NMR data were processed using Topspin 1.3 (Bruker) and visually inspected using Amix 3.8.7 (Bruker, Biospin). ^1^H NMR spectra were obtained by the Fourier Transformation (FT) of the FID (Free Induction Decay), applying an exponential multiplication with a line-broadening factor of 0.3 Hz. The resulting ^1^H NMR spectra were manually phased and baseline corrected using the Bruker Topspin software. Chemical shifts were reported with respect to the residual CHCl_3_ signal set at 7.24 ppm. ^1^H NMR spectra were segmented in rectangular buckets of fixed 0.04 ppm width and integrated, using the Bruker Amix software. The spectral region between 7.50 and 6.95 ppm was discarded because of the peak due to residual protic chloroform at 7.24 ppm. The remaining buckets in the range 9.5–0.02 ppm were then normalized to the total area to minimize small differences due to total olive oil concentration and/or acquisition conditions between samples and subsequently mean-centered. The data set was made of the ^1^H NMR spectra bucket values (columns) measured for the above numbered olive oil samples (rows). Column Pareto scaling was performed (scaling of buckets). In the Pareto scaling a variable is divided by the square root of its variance. This does not eliminate high variance entirely, which may be undesirable, but it gives variables with lower variances a better chance to be detected by PCA [21].

## 3. Results and Discussion

### 3.1. Characterization of the Fatty Acid Content in EVOO

The fatty acid composition of the reference EVOO was evaluated by ^1^H NMR spectra analysis. Data indicated that all the investigated olive oils had the expected EVOO fatty acid composition, with oleic acid being the most abundant for all the cultivars (Table 2 and Table 3). 

**Table 2:** Fatty acid composition (%) of the European and non-European extra virgin olive oil (EVOO) samples obtained from single cultivar and determined by ^1^H-NMR spectral analysis (I: Italy, o: Ogliarola, c: Coratina; S: Spain, Picual; G: Greece, Kalamata; T: Tunisia, Sfax).

Sample	Oleic acid (%)	Saturated Fatty Acids (%)	Linoleic Acid (%)	Linolenic Acid (%)
I-o1	80.44	12.41	8.10	0.97
I-o2	80.83	12.26	7.83	0.93
I-o3	80.64	12.32	8.01	0.98
I-o4	80.52	12.38	8.00	0.92
I-o5	80.77	12.23	7.94	0.97
I-c6	80.05	12.76	8.11	0.94
I-c7	80.08	12.74	8.10	0.93
I-c8	80.10	12.75	8.08	0.95
I-c9	79.96	12.80	8.18	0.96
I-c10	80.18	12.71	8.08	1.00
S1	76.41	14.80	9.80	1.03
S2	76.53	14.79	9.72	1.05
S3	76.54	14.74	9.73	1.03
S4	76.51	14.74	9.78	1.04
S5	76.37	14.82	9.80	1.01
G1	80.02	14.06	7.01	1.11
G2	80.03	14.02	7.01	1.08
G3	80.16	13.96	6.92	1.06
G4	80.01	13.95	7.08	1.06
G5	79.77	14.09	7.23	1.10
T1	68.59	17.73	14.55	0.89
T2	68.72	17.63	14.53	0.90
T3	68.83	17.61	14.46	0.91
T4	68.52	17.74	14.61	0.89
T5	68.54	17.82	14.54	0.92

**Table 3:** Fatty acid composition (%) of blend EVOO samples commercially available as Italian products in the U.S. market. The percentage was determined by ^1^H-NMR spectral analysis.

Cultivar	Oleic acid (%)	Saturated Fatty Acids (%)	Linoleic Acid (%)	Linolenic Acid (%)
M1	78.82	14.03	6.08	1.06
M2	79.77	13.74	5.48	1.00
M3	79.67	13.87	5.38	1.08
M4	75.09	15.98	7.91	1.01
M5	72.23	17.22	9.57	0.98
M6	77.30	14.13	7.55	1.02
M7	68.76	18.72	11.49	1.04
M8	78.05	14.89	5.98	1.08
M9	79.76	13.92	5.37	0.94
M10	79.94	13.06	6.04	0.97
M11	80.35	12.75	5.96	0.94
M12	80.28	12.82	5.97	0.94

### 3.2. European and non-European Single Cultivar EVOO Reference Samples—Principal Component Analysis

**Figure 1:**
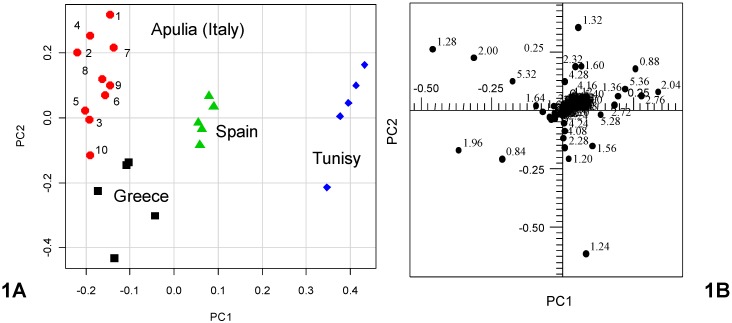
Scatterplot (**A**) and loadings plot (**B)** of the first two PC function scores. PC1 and PC2 explained 46.69% and 30.02% of the total subset variance, respectively.

In the first step, PCA was performed on 25 European and non European olive oils obtained from single cultivar. Coratina (I-c) and Ogliarola (I-o) were chosen as Italian single cultivars, because they are among the most diffused cultivars in the South of Italy and the most used for Italian EVOO production [14]. The reference data set was obtained from 224 bucket-reduced ^1^H NMR spectra (columns values) measured for the 25 reference olive oil samples (rows values) and analyzed by PCA. Relationships between samples and their distribution in groups were defined through the inspection of the resulting scatter plot where each sample was represented by a point in the multidimensional space defined by the principal components. The coordinates of the samples in the new space are called scores. The scatterplot matrix (graphics defined by the axis PC1 *vs.* PC2, or PC3 *vs.* PC4) was observed, in order to investigate similarities and correlations or differences (possible outliers) among the samples, without making any *a priori* assumption. In general, principal components can be evaluated on the basis of the proportion of total variance explained. For the reference olive oil samples, the first four principal components (PC1–PC4) explained 94.12% of the total variance. Nevertheless, for graphical reasons, it seemed to be convenient to consider just the first two components since they explained together 76.71% of the total variance (PC1 46.69%, PC2 30.02%). The resulting scatter plot of PC1 *vs.* PC2 is shown in Figure 1A. It is interesting to observe the dispersion of samples in the bidimensional plane formed by the two first PCs and the arrangement of samples in subgroups (Table 4). The samples identified by the label Italy had negative values of PC1 and essentially positive values of PC2, in particular in the range between −0.25 and −0.15 (for PC1) and −0.15 and 0.30 (for PC2). The samples identified by the label Greece had both negative values of PC1 and PC2, in particular in the range between −0.20 and −0.05 PC1 and −0.50, −0.15 PC2 values. The third group of samples (Spanish oils) was characterized by positive values of PC1 (range between 0.05 and 0.10) and positive and negative values of PC2 (range between −0.1 and 0.1). The last group (Tunisian olive oils) had higher positive PC1 (range between 0.30 and 0.50) and positive and negative PC2 values (range between −0.25 and 0.20) and formed a well-separated cluster. It should be noted that the Italian and Greek samples were very well differentiated from the other two groups on the first principal component (PC1), while they were separated among them on the principal component PC2 (Italian samples for positive and Greek samples for negative values of PC2). Further details arose from the inspection of the PC loadings of the first two PCs. The loadings give the weights of the original variables in the PCs. A high loading indicates a strong contribution of the original NMR signal to the considered PC (indeed the loadings report on the position of each sample within the PC plot). The loading plot is reported in Figure 1B. Examination of the loadings (contributing greatly to PC1 and PC2) suggests that Italian and Greek samples were characterized by a higher amount of oleic acid (δ = 5.32. δ = 2.00. δ = 1.96. δ =1.28 ppm) with respect to Spanish and Tunisian samples, which, in turn, were characterized by a higher amount of saturated (δ = 1.24 ppm) fatty acids. Interestingly, these observations suggest that both the two groups, formed by Italian Coratina and Ogliarola and Greek Kalamata cultivars, contained the highest amount of unsaturated fatty acids and the lowest of saturated fatty acids among the studied reference samples. On the contrary, Sfax and Picual cultivars were characterized by the lowest unsaturated and highest saturated amounts of fatty acids. Moreover, Greek samples contained a higher amount of linolenic acid and a lower amount of linoleic acid with respect to the Italian samples, which results in their differentiation on PC2.

**Table 4:** Dispersion of samples in the bidimensional plane (PC1 *vs*. PC2). Ranges of values are shown.

Samples	PC1	PC2
Italy	−0.25; −0.15	−0.15; 0.30
Greece	−0.20; −0.05	−0.50; −0.15
Spain	0.05; −0.10	−0.10; 0.10
Tunisia	0.30; 0.50	−0.25; 0.20

### 3.3. Blend EVOO Samples Commercially Available as Italian Products in the U.S. Market—Principal Component Analysis

**Figure 2:**
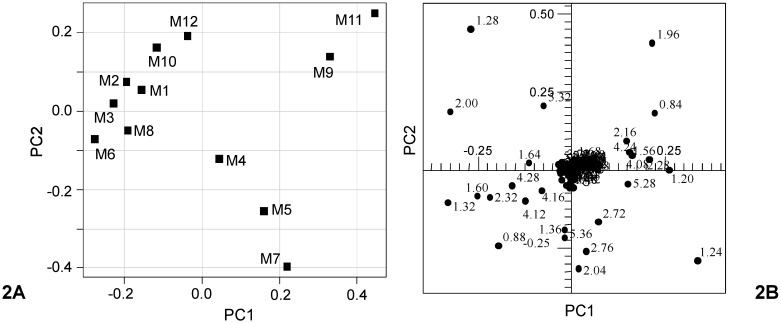
Scatterplot (**A**) and loadings plot (**B**) of the first two PC function scores. PC1 and PC2 explained 51.04% and 32.44% of the total subset variance, respectively.

PCA was performed on 12 EVOO blends commercially available as Italian products in the U.S. The matrix data set was made from 224 bucket-reduced ^1^H NMR spectra (columns values) measured for the 12 blend olive oils (rows values). For the test olive oil samples, the first four principal components (PC1–PC4) explained 95.99% of the total variance. The first two components explained together 83.48% of the total variance (PC1 51.04%, PC2 32.44%). The resulting scatterplot of PC1 *vs.* PC2 is shown in Figure 2A. It is interesting to observe the dispersion of samples in the bidimensional plane formed by the first two PCs and the arrangement in three subgroups (Table 5). The first subgroup (seven samples, M1, M2, M3, M6, M8, M10 and M12) had negative values of PC1 and negative and positive values of PC2, in particular in the range between −0.30 and 0.00 (for PC1) and −0.10 and 0.30 (for PC2). The second group, including two samples (M9 and M11), had positive values of both PC1 and PC2, in particular in the range between 0.30 and 0.45 (for PC1) and 0.10 and 0.30 (for PC2). The last group, formed by three samples (M4, M5, M7), had positive values of PC1 (range between 0.00 and 0.25) and negative values of PC2 (range between −0.40 and −0.10). The loadings plot is reported in Figure 2B. Examination of the loadings (contributing greatly to PC1 and PC2) suggested that seven samples (M1, M2, M3, M6, M8, M10 and M12) were characterized by a higher amount of unsaturated acids (in particular oleic and linoleic, corresponding chemical shifts δ = 5.32, δ = 2.00, δ = 1.28 ppm). On the other hand, separation of samples on PC1 between the group with seven and two (M9, M11) samples can be ascribed to the decrease of linoleic acid and increase of linolenic with an intermediate position for the three blend samples (M4, M5 and M7). Arrangement at lower PC2 values of the group formed by three samples (M4, M5, M7) in the scatterplot was due to higher saturated fatty acids (δ = 1.24 ppm). It should be noted that the PC1 differentiation of samples in the scatterplot of Figure 2A was achieved due to loadings related to signals which contribute to PC2 in the scatterplot of Figure 1A.

**Table 5:** Dispersion of the blend EVOO samples in the bidimensional plane (PC1 *vs*. PC2). The value ranges are shown.

Samples	PC1	PC2
M1, M2, M3, M6, M8, M10, M12	−0.30; 0.00	−0.10; 0.30
M9, M11	0.30; 0.45	0.10; 0.30
M4, M5, M7	0.00; 0.25	−0.40; −0.10

### 3.4. Comparison between the Blend EVOO Samples Commercially Available as Italian Products in the U.S. Market and the Olive Oils Obtained from European and Non-European Single Cultivar Reference Samples—Principal Component Analysis

**Figure 3:**
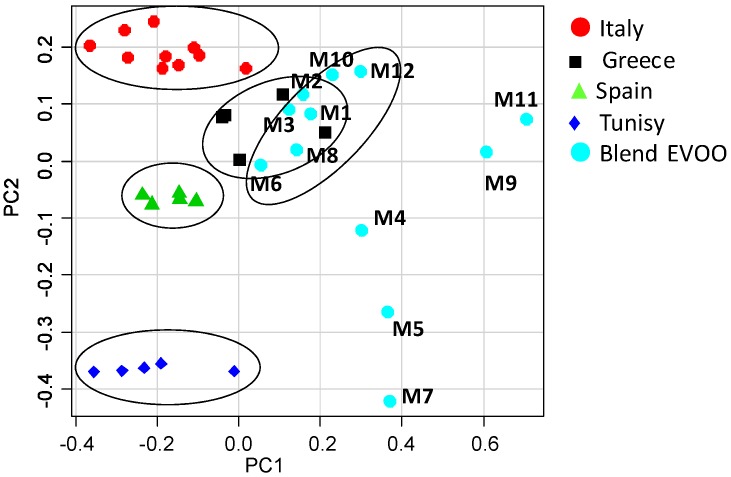
Scatterplot of the first two PC for the whole olive oil dataset. PC1 and the PC2 explain 49.09% and 29.26% (together 78.35%) of the total variance, respectively.

The whole data set of 224 bucket-reduced ^1^H NMR spectra (columns) for 37 EVOO samples (rows: 25 reference samples and 12 blends commercially available as Italian products in the U.S.), was analyzed by PCA. A clear discrimination between samples of different cultivars and geographical origin was revealed. Since the cultivars of the examined blends were unknown, discussion related to the blend olive oils has been limited only to metabolites composition. The olive oil samples were distributed in the PC1/PC2 scatterplot according to their intrinsic features and to their major/minor affinity in comparison with the reference samples included in this case study. The first four principal components (PC1–PC4) explained 92.43% of the total variance. The principal components PC1 and PC2, shown in the Figure 2 scatterplot, explained 78.35% of the total variance (PC1 49.09%, PC2 29.26%) while PC3 (8.41%) and PC4 (5.67%) accounted for the residual variance. The PC1/PC2 scatterplot showed that distinct clusters were formed for the studied samples (Figure 3). A separation between the mono cultivar reference olive oils and the U.S. commercially available blends was observed. In particular, the PC1 axis separated blends, in the range between 0.05 and 0.70, from the reference samples, in the range between −0.45 and 0.05, with the exception of the Greek references, which were in the PC1 range between −0.05 and 0. 20. Italian olive oils were nearly all included in the range between 0.05 and −0.40 for PC1; Spanish samples between −0.30 and −0.10 for PC1, and Tunisian samples between −0.40 and 0.00 for PC1. Moreover, from the PC2 comparative analysis a good affinity of most of the blend samples was found with the European olive oils (Spain, Greece and Italy). The PC2 highlighted differences among the reference samples since the samples were well distributed along the PC2 axis (Tunisians between −0.35 and −0.40, Spanish −0.50 and −0.10, Greek 0.00 and 0.15 and Italian olive oils 0.15 and 0.25). Two subgroups were observed for the commercial blends along the PC2 axis, the largest of them (9 samples) shared the same European PC2 range (between −0.05 and 0.20) while the second group (3 samples) fell at a PC2 range closer to the Tunisian references (between −0.10 and −0.45). It should be noted that 7 out of 9 blend samples were much closer to the European reference oils also on the PC1 (range between 0.00 and 0.40) and nearly merged with the Greek reference samples (Table 6). 

**Table 6:** Dispersion of samples in the bidimensional plane (PC1 *vs.* PC2). Ranges of values are shown.

Samples	PC1	PC2
Italy	−0.40; 0.05	0.15; 0.25
Spain	−0.30; −0.10	−0.05; −0.10
Greece	−0.05; 0.20	0.00; 0.15
Tunisia	−0.40; 0.00	−0.40; −0.35
M1, M2, M3, M6, M8, M10, M12	0.05; 0.40	−0.01; 0.20
M9, M11	0.60; 0.70	0.00; 0.10
M4, M5, M7	0.30; 0.40	−0.45; −0.10

In summary, the examined commercial blends could be divided into three groups: the first (7 samples) shared similar PC1 and PC2 ranges with European references (in particular Greek EVOO); the second (2 samples) displayed the same PC2 values of European samples but higher PC1 values; the third (3 samples) showed the same PC1 values as the second but PC2 values more similar to those of the Tunisian samples (especially for M5 and M7). Therefore, the higher similarity of the studied blend oils was detectable for European reference samples, in particular from Greece, Italy and Spain. It is interesting to note that the Greek reference samples were found just inside the bidimensional space (PC1, PC2) comprising the group of 7 out of 12 examined oils. Comparison of Figure 3 with Figure 1A shows that the introduction of the blend samples in the data set resulted in a distribution of the reference groups where the PC1 and PC2 have been interchanged. This may be due to the presence in the blend samples of considerable amounts of the same metabolites that differentiated Italian from Greek olive oils in Figure 1. Indeed PC1 differentiation of samples in the scatterplot of Figure 2A is due to loadings related to signals which contribute to PC2 in the scoreplot of Figure 1A. Examination of the loadings suggests that the separation of the nine blend samples having PC2 > −0.1 (M1, M2, M3, M6, M8, M9, M10, M11 and M12), which include the seven very close to the European also in terms of PC1 (M1, M2, M3, M6, M8, M10 and M12) was achieved due to spectral domains belonging to the resonances of acyl groups of oleic, linoleic and linolenic chains (δ = 1.28, 1.96, 2.00, 5.32 ppm). On the contrary, the group of the residual three samples (M4, M5, M7), although with higher PC1 values, were closer to the Tunisian samples due to higher saturated (δ = 1.24 ppm) fatty acids (M7 > M5 > M4) [12]. Separation of samples on PC1 in the plot of Figure 3 can be ascribed to the decrease of linoleic acid and increase of linolenic, which can be observed on going from Italian to Greek samples as well as from the seven (M1, M2, M3, M6, M8, M10 and M12) to the two (M9 and M11) blend samples with an intermediate position for the three high saturated blend samples (M4, M5 and M7). In general, blend olive oils are characterized by a higher amount of polyunsaturated fatty acids and a lower amount of saturated fatty acids than single cultivars samples. These results agree with previous findings suggesting that commercial olive oils could be distinguished according to their geographical origin depending on their major components (triglycerides). 

The triglyceride composition was found to be particularly useful in discriminating the oil samples. In comparing the studied varieties, small but significant differences among the samples were shown; indicating a slight varietal effect on fatty acid composition. Furthermore, as the fatty acid composition is an important quality parameter and authenticity indicator of EVOO, these results are in agreement with other studies in which nutritional properties of EVOO have been described [22]. The high content of monounsaturated, low saturated and linoleic fatty acids, which characterize in particular some of the studied samples, the European EVOO and, among them, the Italian EVOO, is of great importance because of their high nutritional value and positive effect on the oxidative stability of oils [23]. 

## 4. Conclusions

Chemometric techniques (unsupervised PCA) were applied to the ^1^H NMR data of blend extra virgin olive oils commercially available in the U.S., as Italian products, and compared with monovarietal extra virgin olive oils from Italy, Greece, Spain and Tunisia. Results show that the spectral data contain useful information for the geographical characterization of samples. Moreover, our preliminary study of the ^1^H NMR spectra of monovarietal oils from four different countries suggests that the spectra contain information which could help the classification of blends according to their geographical origin. In general, some trends related to the geographical origin of the commercially blend olive oils are observed. U.S. blend samples M4, M5, M7, M9 and M11 are “clustered” in the PC scatterplot in two groups far away from all monovarietal olive oils included in this study according to PC1 (M11, M9) or both PC1 and PC2 (M4, M5, M7). All the other U.S. commercialized olive oils, declared on the label as Italian, are closer to Greek (in particular) and Spanish oils rather than to the Italian monovarietal oils. However, the latter originated from few (although most popular) olive cultivars of Apulia (the leading Italian producer region) and not from other Italian regions. It should be, therefore, pointed out that the blends sold in the U.S., and used in this study, are different from the standard Italian monovarietal oils included for comparison, but still could be similar to other Italian olive oils. Thus, this paper only describes the potentiality of the approach, since a more complete database of standards must be used to gain further evidence for the origin of the analyzed olive oils. 

Fatty acid composition affects the quality and also the taste of EVOO, a condiment which is largely responsible for healthy effects of the Mediterranean diet. Moreover, it has been proven that the fatty acid content can contribute to an olive oil’s characterization since it is known that its acidic profile is mainly affected by the fruit variety and geographical origin. The findings of this work reveal that the application of PCA may be an optimal solution for the evaluation of EVOO traceability by both geographical and botanical points of view. In fact, good resolution among blend olive oils commercially available in the U.S. with respect to other single cultivar of EVOO such as Coratina and Ogliarola from Italy (Apulia), Picual from Spain, Kalamata from Greece and Sfax from Tunisia was found. The availability of a larger data set of genetically certified monovarietal extra virgin olive oils would further facilitate the classification of commercial olive oils and also provide a way to guarantee the mandatory labeling reporting the geographical origin of olive oils as suggested in the last EU Regulation 182/2009 [24].

## References

[1] Covas M.I., Nyyssönen K., Poulsen H.E., Kaikkonen J., Zunft H.J., Kiesewetter H., Gaddi A., de la Torre R., Mursu J., Bäumler H. (2006). The effect of polyphenols in olive oil on heart disease risk factors. A randomized trial. Ann. Intern. Med..

[2] Grigg D. (2001). Olive oil, the Mediterranean and the world. GeoJournal.

[3] Ogrinc N., Košir I.J., Spangenberg J.E., Kidrič J. (2003). The application of NMR and MS methods for detection of adulteration of wine, fruit juices, and olive oil. A review. Anal. Bioanal. Chem..

[4] Temime S.B., Taanalli W., Baccouri B., Abaza L., Daoud D., Zarrouk M. (2006). Changes in olive oil quality of Chétoui variety according to origin of plantation. J. Food Lipids.

[5] Paz Aguilera M., Beltran G., Ortega D., Fernandez A., Jiménez A., Uceda M. (2005). Characterization of virgin olive oil of Italian olive cultivars “Frantoio” and “Leccino”, grown in Andalusia. Food Chem..

[6] D’Imperio M., Gobbino M., Picanza A., Costanzo S., Della Corte A., Mannina L. (2010). Influence of harvest method and period on olive oil composition: An NMR and statistical study. J. Agric. Food Chem..

[7] Papadia P., Del Coco L., Muzzalupo I., Rizzi M., Perri E., Cesari G., Simeone V., Mondelli D., Schena F.P., Fanizzi F.P. (2011). Multivariate analysis of ^1^H NMR spectra of genetically characterized extra virgin olive oils and growth soil correlations. J. Am. Oil Chem. Soc..

[8] Vlahov G., Del Re P., Simone N. (2003). Determination of geographical origin of olive oils using ^13^C nuclear magnetic resonance spectroscopy. I. Classification of olive oils of the Puglia region with denomination of protected origin. J. Agric. Food Chem..

[9] Giansante L., Di Vincenzo D., Bianchi G. (2003). Classification of monovarietal Italian olive oils by unsupervised (PCA) and supervised (LDA) chemometrics. J. Sci. Food Agric..

[10] Muzzalupo I., Stefanizzi F., Perri E. (2009). Evaluation of olives cultivated in Southern Italy by SSR markers. HortScience.

[11] International Oliver Council. http://www.internationaloliveoil.org.

[12] Alonso-Salces R.M., Holland M.V., Guillou C. (2011). ^1^H-NMR fingerprinting to evaluate the stability of olive oil. Food Control.

[13] Godini A. (2011). Olive cultivars field-tested in super-high-density system in southern Italy. Calif. Agric..

[14] Sarnari T., Monduzzi F., Carbonari F. (2010). Stime produttive per la campagna olearia 2010/2011. http://www.ismea.it/flex/cm/pages/ServeAttachment.php/L/IT/D/D.93f782233e547d7be4ce/P/BLOB%3AID%3D5548.

[15] Montalbano Agricola Alimentare Toscana S.p.A. http://www.oliomontalbano.it/.

[16] Azienda Olearia Basile s.n.c. http://www.aziendaoleariabasile.com.

[17] Mannina L., Dugo G., Salvo F., Cicero L., Ansanelli G., Calcagni C., Segre A. (2003). Study of the cultivar-composition relationship in sicilian olive oils by GC, NMR, and statistical methods. J. Agric. Food Chem..

[18] Guillen M.D., Ruiz A. (2003). Rapid simultaneous determination by proton NMR of unsaturation and composition of acyl groups in vegetable oils. Eur. J. Lipid Sci. Technol..

[19] Mannina L., Sobolev A.P. (2011). High Resolution NMR characterization of olive oils in terms of 379 quality, authenticity and geographical origin. Magn. Reson. Chem..

[20] R Development Core Team (2009). R: A Language and Environment for Statistical Computing.

[21] Blekherman G., Laubenbacher R., Cortes D.F., Mendes P., Torti F.M., Akman S., Torti S.V., Shulaev V. (2011). Bioinformatics tools for cancer metabolomics. Metabolomics.

[22] Dabbou S., Dabbou S., Selvaggini R., Urbani S., Taticchi A., Servili M., Hammami M. (2011). Comparison of the chemical composition and the organoleptic profile of virgin olive oil from two wild and two cultivated Tunisian *Olea europaea*. Chem. Biodivers..

[23] Diraman H., Dibeklioglu H. (2009). Characterization of Turkish virgin olive oils produced from early harvest olives. J. Am. Oil Chem. Soc..

[24] The Commission of the European Communities (2009). Commission Regulation (EC) No 182/2009 of 6 March 2009 amending Regulation (EC) No 1019/2002 on marketing standard for olive oil. Off. J. Eur. Union.

